# Combined Additive and Laser-Induced Processing of Functional Structures for Monitoring under Deformation

**DOI:** 10.3390/polym15020443

**Published:** 2023-01-14

**Authors:** Tawakalt Mayowa Akintola, Balaji Krishna Kumar, Tarik Dickens

**Affiliations:** 1 Industrial & Manufacturing Engineering, FAMU-FSU College of Engineering, 2525 Pottsdamer St., Tallahassee, FL 32310, USA; 2 High-Performance Materials Institute, 2005 Levy Ave., Tallahassee, FL 32310, USA

**Keywords:** laser-induced graphene, laser processing, additive manufacturing, fused deposition model

## Abstract

This research introduces a readily available and non-chemical combinatorial production approach, known as the laser-induced writing process, to achieve laser-processed conductive graphene traces. The laser-induced graphene (LIG) structure and properties can be improved by adjusting the laser conditions and printing parameters. This method demonstrates the ability of laser-induced graphene (LIG) to overcome the electrothermal issues encountered in electronic devices. To additively process the PEI structures and the laser-induced surface, a high-precision laser nScrypt printer with different power, speed, and printing parameters was used. Raman spectroscopy and scanning electron microscopy analysis revealed similar results for laser-induced graphene morphology and structural chemistry. Significantly, the 3.2 W laser-induced graphene crystalline size (La; 159 nm) is higher than the higher power (4 W; 29 nm) formation due to the surface temperature and oxidation. Under four-point probe electrical property measurements, at a laser power of 3.8 W, the resistivity of the co-processed structure was three orders of magnitude larger. The LIG structure and property improvement are possible by varying the laser conditions and the printing parameters. The lowest gauge factor (GF) found was 17 at 0.5% strain, and the highest GF found was 141.36 at 5%.

## 1. Introduction

In advanced manufacturing, 3D printing has gained a lot of attention in the last decade. Due to the versatility in the materials, designs, and ease of custom tailoring, products and performing topographies could not be produced using conventional subtractive manufacturing [1,2]. In recent times, extensive research has achieved fully functional 3D printed structures, where the modern integration of conductive systems components is of future relevance in the fabrication of sensors, circuits, 3D electronics, antennas, etc. [3,4]. Along with this, polymer composites have demonstrated distinctive properties based on their design [5,6,7], strength [8], and thermal and electrical conductivity [3,9]. Thus, it is a material of interest for various applications such as those in the aerospace, automobile, and oil and gas industries, as well as for electronics components. Apart from the materials, various additive manufacturing (AM) techniques have been introduced and involved effectively; however, fused deposition modeling (FDM) [10,11,12] described the prominent compatibility with regard to electronic component fabrication [13,14].

Significantly, thermoplastic polymer utilization in a fused deposition model (FDM) demonstrated rapid, simple fabrication at an affordable cost; however, the final product could have been executed with poor mechanical, electrical, and thermal properties. Due to these reasons, the current studies have adequately introduced thermoplastics, which exhibit the prevailing mechanical, electrical, thermal, and required properties. However, introducing nanofiller in the polymer matrix has been considered to be a complex process under different conditions, where the major causes, such as adhesion, orientation, and dispersion of the nanomaterials, constrain it. Consequently, this also affects printing parameters and appropriate printing structures.

Nowadays, several studies have been introduced with laser-induced techniques; similarly, laser-induced graphene (LIG) has been used for multifunctional applications. Reportedly, the use of the laser process offers a top-down, noncontact method that produces a graphene-like structure through the laser irradiation process [15]. For instance, Rahimi et al. [16,17] and Luo et al. [18] reported work focusing on laser power and the beam scan speed-changing effect in the end formation. Ye et al. [19] described that the regulation of (i) laser parameters, (ii) atmosphere, and (iii) substrate to control the porosity, composition, and morphology would impact the chemical, physical, and electronic properties of derived LIG. This controllable approach towards synthesizing LIG and their significant electrical properties have made them versatile materials for various applications. Hence they have been utilized in laboratory and commercial areas, including the development of flexible electronics [20,21,22], biodegradable devices [23], chemical detection [19,24], sensors for sound [25,26,27], and microfluidics [28,29]. Altogether, the involvement of LIG in specified fields required particular properties of interest. The ability to create unique designs and a layering orientation with high resolution is visible in additive manufacturing (AM), and laser processing has been shown to offer a top-to-bottom, noncontact, and highly selective one-step method that produces graphene with highly unique properties. Additionally, it explores co-fabrication methods that are helpful to improve the manufacturability/design, repeatability, and scalability of PEI thermoplastic materials to achieve laser-induced graphene (LIG) with electrical properties, which can be used as a printed flexible sensor.

Recently, Tavakkoli et al. reported a similar kind of investigation with a single laser pass, and the study was performed with 10–40% of laser power from a 30 W laser (the pulse-width modulation was dependent on the average power; thus, the spacing between the pulses was 1000 dots per inch (DPI). The laser spot size was 76 μm, and the scan rate was 2.54 cm/s). All samples indicate D and G bands at 1300 and 1550 cm^−1^, respectively. However, the intensity peaks for performed laser power samples at 10, 20, and 30% were not very high. Moreover, the D and G bands were connected, corresponding to high-temperature annealed nano graphite. The best result was achieved at 40% laser power, which was used to determine the piezo resistivity of the strain sensor [30]. In this work, we utilize a low power range of 3.2–4.0 W, a constant laser scan rate of 38.1 mm/s, and multiple scan times to derive LIG with fewer defects. All samples have a high D/G peak intensity and fewer defects, and the highest degree of graphitization was derived at a lower power of 3.2 W. The use of multi-lasing indicates fewer defects in the graphitic structure, because laser irradiation relies on the accumulation of multiple irradiations with lower fluencies to achieve high-quality graphitic structures consistently [31], as opposed to single-lasing with loosely packed fluff prone to losing the load transfer efficiency in the substrate. Moreover, this study details how the process–structure–property relationship changes according to the complicated laser process, which is helpful in estimating the formation of and controlling the LIG production for definite needs. Apart from that, the investigation of laser scribing was performed on the FDM 3D printed Ultem (polyetherimide PEI) sample surface. Due to additive manufacturing, PEI’s toxic chemical production steps were diminished; the proper construction of the sample would enhance the uniform production of graphene after certain laser scribing on the material. The conductive graphene on the AM parts could interconnect with multifunctional properties. Thus, the integration of LIG and additive manufacturing was achieved.

## 2. Material and Methodology

### 2.1. Preparation of LIG by 3D Printing and Laser Processing

A high-precision 3Dn-300 nScrypt printer fed with the Ultem filament with 1.75 diameters (PEI, purchased from McMaster Carr) extruded via an nFD material extrusion tool head was used to fabricate all specimens in this study (Table 1). Before printing, a PEI filament was placed in the oven overnight at 50 °C to dry out the moisture, and the printed specimen dimensions (L × W × T) were 60 mm × 20 mm × 0.3 mm, where the layer was ‘n = 1′. In addition, the building direction was fixed along the edge (X–Z) axis to attain a better surface finishing.

Initially, the printing temperature of the nozzle (350 μm) and the bed were kept at 390 °C and 160 °C, respectively. A laser engraving and cutting system (Versa Laser VL-300, Universal Laser System, Inc., Scottsdale, AZ, USA) equipped with a CO_2_ laser with a wavelength of 10.6 μm m was helpful in generating the graphitic sensor arrays through the irradiation on the printed specimen film. The beam size of the CO_2_ laser was ~100 μm, and the thickness of the specimen was 0.3 mm. The laser beam inscribed lines on the printed surface during the process, which was designed via CorelDRAW X3 graphic design software. Pulse per inch or pixel per inch (PPI) and laser speed were fixed at 500 PPI and 38.1 mm/s, respectively. Furthermore, the power (3.2–4.0 W +/− 0.2) was varied effectively to understand the laser-power-dependent changes. Hence, the changes in morphology, electrical properties, and heat transfer were investigated; however, the laser parameter was selected based on relevant studies [27,32].

### 2.2. Characterization Techniques

The Raman spectroscopy (Renishaw inVia Raman microscope) analysis was performed with a 633 nm excitation laser at 1 mW. In addition, Ultraviolet–visible (UV-Vis) absorbance and transmission were measured using the UV-5000 UV-Vis NIR spectrophotometer (Varian). Significantly, the crystalline size along the axis (*L_a_*) was calculated from the utilized Raman wavelength and the ratio of the integrated intensity G/D peaks. Equation (1) was used to obtain *L_a_*:

(1) $$L_{a}=\left(2.4\times 10^{-10}\right)\times \lambda_{l}^{4}\times \left(\frac{I_{G}}{I_{D}}\right).$$

The wavelength used in this study (λ) is 633 nm.

Then formed graphitic line features’ (based on the CO_2_ laser irradiation) morphological structures were examined with scanning electron microscopy (SEM) (Phenom XL Desktop SEM at 10 kV). Probes were employed to evaluate the resistance of the LIG lines recorded by a multimeter. The resistivity was calculated using the cross-sectional values of the LIG from SEM and the length of the graphitic line. For all tests, a silver paste (Electron Microscopy Sciences) and a gold conductive tape were applied on the edge of the sensor for connection then cast with an epoxy resin layer onto the LIG surface for compaction to avoid delamination from the PEI surface. Further, a cyclic three-point bending test was conducted on the sample using an AGS-X mechanical test machine (500 N load cell, Shimadzu Scientific Instruments, Inc, Columbia, MD, USA), and the following settings were used for the test in detail: gauge length = 20 mm; displacement rate 4 mm/s; and cyclic strain 0.5, 1, 3, and 5. At the same time, the change in resistance of the mechanically deformed sensor was recorded by a Keithley 2401 source meter controlled by a homemade LabView user interface. Furthermore, the gauge factor was used to investigate the sensitivity of the LIG-PEI corresponding to the strain, the gauge factor (Equation (2)),

(2) $$GF=\frac{1}{R_{0}}\frac{R_{max}-R_{0}}{\varepsilon_{m}},$$

where the $\varepsilon_{max}$ is the amplitude of strain applied in the cyclic bending test, $R_{max}$ is the sensor resistance when the applied strain is at $\varepsilon_{max}$, and $R_{0}$ is the sensor resistance without the deformations used.

### 2.3. Laser Processing and Mechanism

**Figure 1:**
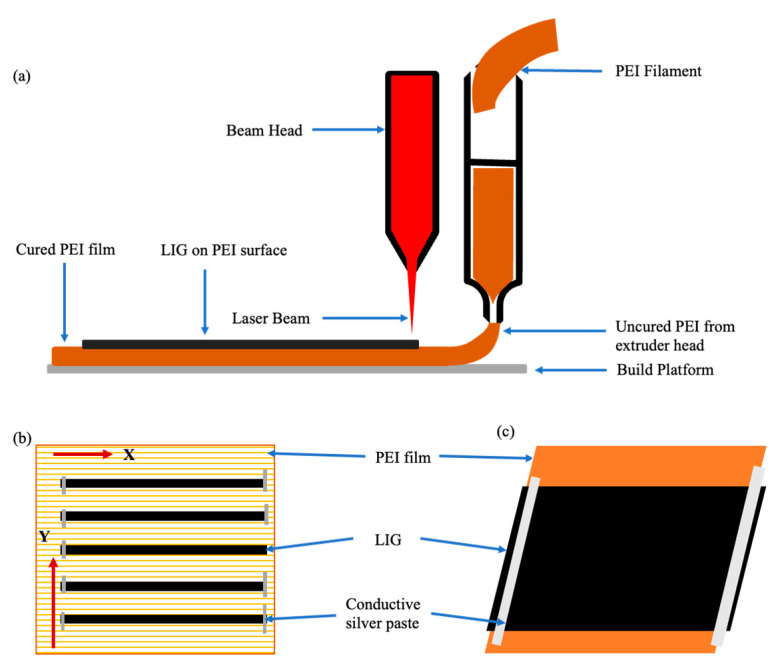
Schematic image of (**a**) laser processing, (**b**) five processed lines with four laser passes each, and (**c**) printed sensor, approximately 60 mm in length. The laser was passed across the Y-direction at 90° orientation in line with the printed PEI material in this processing configuration.

After successfully printing the PEI film via nScrypt, the formed film was kept in a Versa CO_2_ laser source, which could convert the surface into LIG, using the aforementioned laser processing parameters. The schematic image of the attained outcome is shown sequentially in Figure 1. However, significant laser conditions were achieved based on the optimization of many test samples during this work. Several samples exhibited no surface conversion after allowing changes such as high power, low speed, and unfocused beam lenses in order to result in evident graphitic formations. Therefore, these samples were omitted from the analysis. Five samples were printed and laser-induced in an open-air environment for comparison purposes, and the resulting structure has been referred to as a graphitic structure. A similar process was utilized to generate the printed sensors used for strain gauge testing. A laser with parameters of pulse per inch or pixel per inch (PPI), laser speed, and power of 500 PPI, 38.1 mm/s 3.2 W, respectively, was fixed to obtain the printed sensor.

## 3. Results and Discussion

### 3.1. Raman Spectra

**Figure 2:**
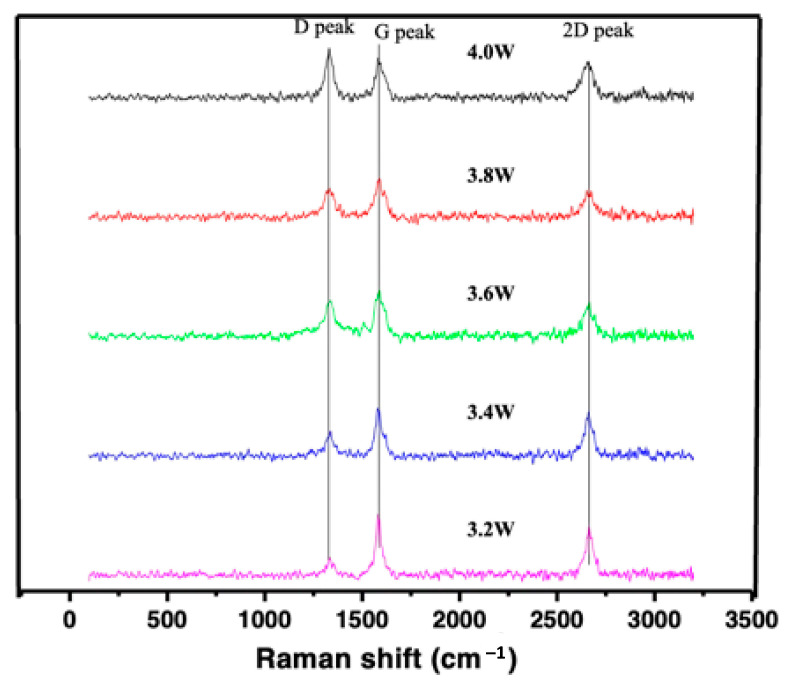
Raman spectra of LIG obtained vs. laser power.

CO_2_ that was laser irradiated on the printed film formed a graphitic structure and was examined using Raman spectroscopy as shown in Figure 2. In addition to the different laser power, the impact in the graphitic formation was estimated via Raman peaks. Different peaks were shown at 1362 cm^−1^ (D-band), 1600 cm^−1^ (G-band), and 2700 cm^−1^ (2D bands). The D-band indicated the sp^3^ atomic state, which denoted few disorders or impurities in the graphene structure; it also suggested an amorphous carbon component [33,34]. Next, the attained G-band determined the carbon–carbon bond stretching, which was an evident formation of the graphene structure. The 2D peaks at 2700 cm^−1^ indicate the few-layered graphene stacks [34]. Overall, these peaks were consistent with already reported results for LIG-PI [15]. LIG derived from PEI through laser irradiation is best described as a graphitic structure to avoid misuse of the term with graphene.

**Figure 3:**
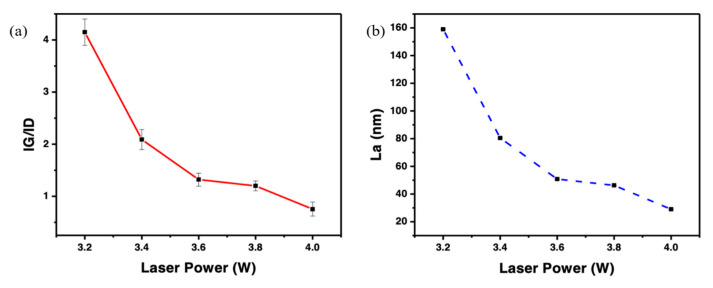
(**a**) Integrated intensities of analysis of G to D peaks (IG/ID) and (**b**) average crystalline size La as a function of laser power.

In addition, the relationship between the laser power and the intensity ratio of the G to D peaks was calculated. Additionally, the statistical analysis of IG/ID was determined at five different points and plotted against the laser power in Figure 3a. The result shows that the highest degree of graphitization was achieved at 3.2 W. Moreover, the further increment of laser powers resulted in a negative impact on LIG formation. This could be due to their oxidation degrading the LIG’s quality [15]. The use of multi-lasing indicates fewer defects in the graphitic structure, as the laser irradiation relies on the accumulation of multiple irradiations with lowered fluencies to achieve high-quality graphitic structures steadily, unlike single-lasing with loosely-packed fluff prone to lose the load transfer efficiency in the substrate [32,35,36]. Beyond Large-Area Bernal-Stacked Bi structures, Figure 3b details the graphene crystalline size (La), calculated from the Raman results using Equation (1). The formed LIG at 3.2 W demonstrated a crystalline size of 159 nm, attributed to changes in the surface temperature [15]. However, enhancing the power reduced the graphene sizes effectively. Thus, the observed La (29 nm) value at 4 W was reduced substantially.

### 3.2. UV-VIS Analysis

**Figure 4:**
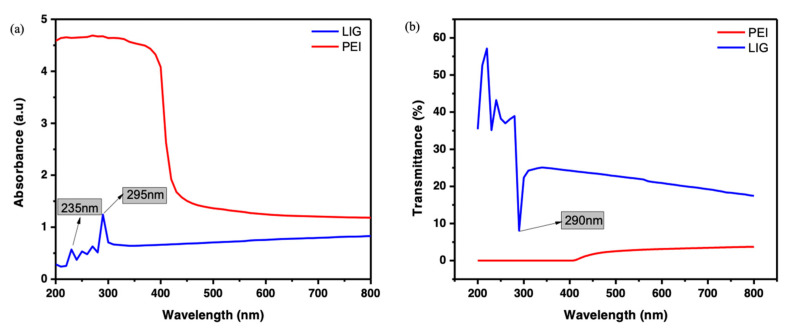
UV-Vis (**a**) absorption and (**b**) transmittance spectra of PEI and LIG.

Here, the UV-VIS analysis was investigated to estimate the light–matter interaction of the printed PEI tab and LIG scraped from the surface, as shown in Figure 4a,b. Generally, PEI shows a broad absorption band in the range of 200 to 400 nm, which could indicate charge–transfer complexes (CTC), which may occur in the form of intermolecular and intermolecular CTC. The PEI absorption band around 200 nm is known to be associated with intramolecular charge–transfer interaction, while 300 to 400 nm are related to intermolecular ones while higher [37,38]. The absorbance spectra of LIG (dispersed in ethanol) exhibited excellent absorption in the visible range of 200 to 300 nm, which is similar to those of graphene [39,40].

Two absorption peaks were noticed in Figure 4a. The first peak was observed at 235 nm, corresponding to the pi-pi transition of the aromatic C-C bonds. The second peak was noted at 295 nm, described as the shifts of the n-pi transition of the C = O bonds [41,42]. Figure 4b shows the PEI and LIG transmittance spectra (ranging from 200–800 nm). The spectra show that the PEI completely blocked the light wavelengths below 400 nm, although it was transparent in the expressed visible region. Meanwhile, LIG indicates an enhanced UV-light shielding capacity in the visible range, which peaks at 295 nm. Even though LIG exhibited no transmittance in the visible range, it possesses an excellent photo-response similar to graphene in the UV range.

### 3.3. Optical Analysis

**Figure 5:**
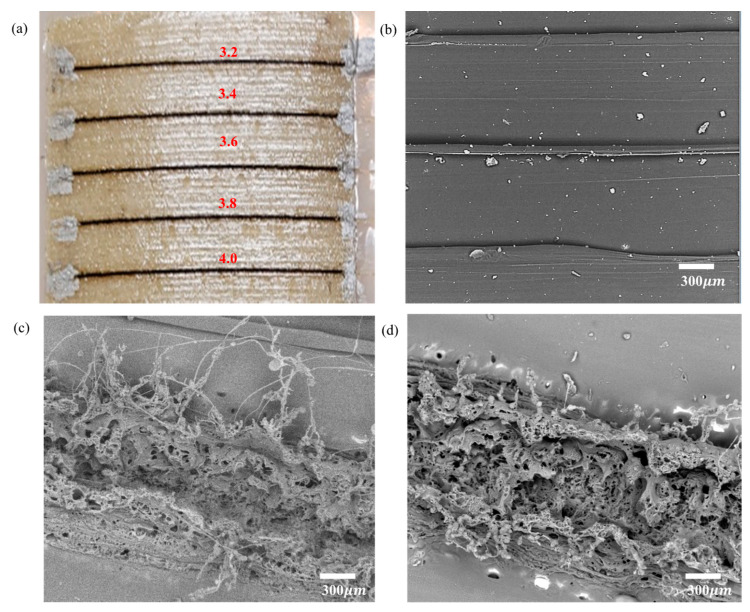
(**a**) Surface image of printed PEI surface converted into LIG at 3.2, 3.4, 3.6, 3.8, and 4 W; SEM images of (**b**) Printed PEI surface without laser power; (**c**,**d**) surface images of 3.2 and 4 W laser power at 300 μm.

**Figure 6:**
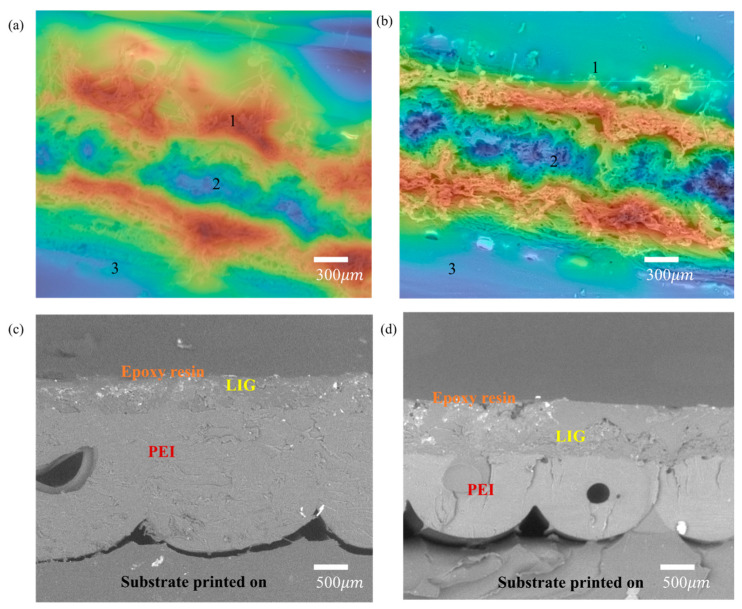
(**a**,**b**) 3D Surface roughness and (**c**,**d**) cross-sectional image of 3.2 and 4 W of laser power at 300 μm.

The optical images in Figure 5a represent the different laser-power-scribed printed PEI samples, and the surface was irradiated through the printing direction. After that, SEM surface images were included to understand their construction and morphological changes across power settings (Figure 5b,d) and surface roughness (Figure 6). Initially, polymer decomposition into aromatic and linear oligomers will occur during the laser irradiation of the PEI. The aromatic ring pyrolysis products were then converted in the LIG (Figure 5c,d) in a high-temperature environment generated by the laser. The lower power 3.2 W laser yielded the highest graphitization by achieving fewer defects and enough surface temperature to ablate and convert from sp3 to sp2 carbon, known as the photothermal process; evidently, this is similar to the low power density graphitization process demonstratable with other materials [43]. To enhance the formation, the laser scribing process was repeated four times to achieve the high level of macropores and lower level of micropores contained in the LIG sheets. Furthermore, it reduced gaseous product generation during the pyrolysis yield [15,27].

The surface roughness was determined by comparing the LIG on the PEI at low and high laser power. Compared to the low power shown in Figure 6a, the high laser power creates a large surface and depth area due to the high-energy laser concentration (Figure 6b). Figure 6 show a thermal image of a surface with color gradient indications labeled 1, 2, and 3 (Figure 6a,b). Label 1 in the figure is the observed peak profile, label 2 is the valley where the concentration of the laser power hit due to the beam’s focus, and label 3 is the surrounding area of the LIG where the effect of the laser power was low with the initial LIG conversion occurrence. The mean roughness of the average distance between the peaks and valleys of the 3.2 to 4 W was measured to be 6.06 μm to 11.41 μm, and this significant difference is due to the laser effect on the PEI. Finally, a cross-sectional image of LIG-PEI is attached in Figure 6c,d, revealing the increased LIG thickness on the PEI surface from 56.3 μm to 220.5 μm (Table 2). In addition, to secure the LIG on the printed PEI, the LIG was coated with liquid epoxy resin, which penetrated the layers and firmly bonded the fluffy porous structure on the PEI substrate. This keeps the LIG layers from falling off as the PEI substrate is moved for further analysis and testing. The average thickness of the LIG and the surface height are listed in Table 2.

### 3.4. Electrical Property

**Figure 7:**
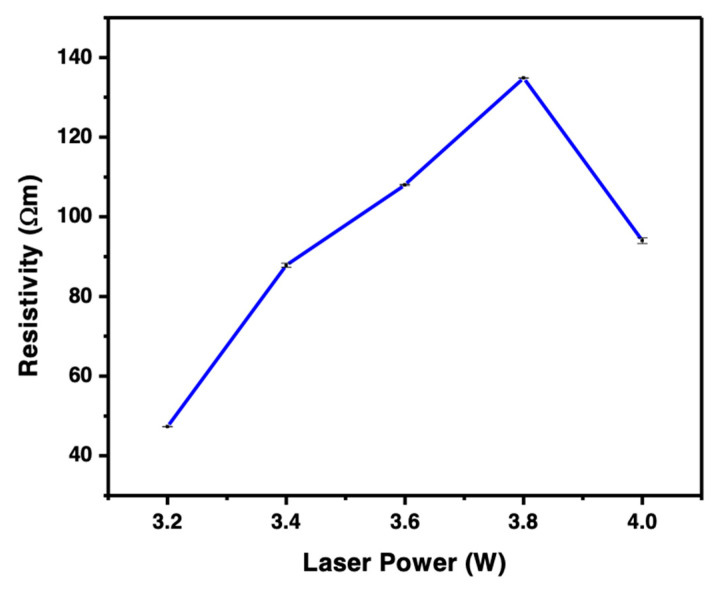
Variation in the resistance of LIG under different CO_2_ laser powers.

The calculated resistivity maintains a slow steady increment as the laser power increases from a low value to 3.8 W, as shown in Figure 7. An increase in the resistivity is attributed to the changes in the cross-sectional area as the laser increases. As the laser power increases to 4 W, the resistivity gradually reduces to 94.1064 Ωm. Based on the literature, the property of the LIG could be affected more at higher laser power than at lower power. This is due to the change in laser power in 4 W that leads to increased oxidation and a deterioration in quality of the LIG. Hence, there is a drop in the resistivity level [15]. Due to the high laser power, the thermal expansion of LIG was observed, which means it was exposed to receive an intensive heat input of laser irradiation, which subsequently affected their surface.

### 3.5. Piezo Resistivity of the LIG Sensor

Altogether, the extensive analysis and property observation of material and additive manufacturing introduces the performed material into sensor application. Owing to that, the potential performance of LIG as a sensor of electronic components was investigated via piezo resistivity tests. The 3D printed LIG-PEI sample was utilized as a strain sensor, measured with the bending of deformed resistance changes. Since the PEI is a plastic material, a plastic deformation was expected, where considerable strain deformation exhibited a variation of the relative resistance change.

**Figure 8:**
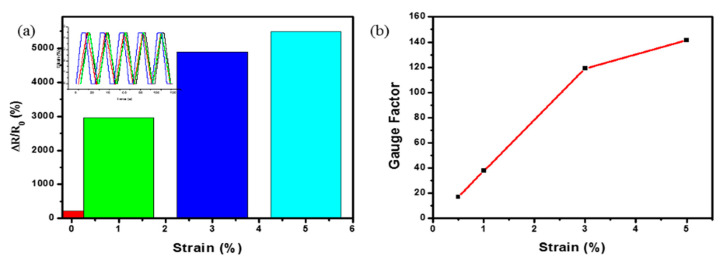
(**a**) Relative resistance change (ΔR/Ro) of the LIG-PEI sensor vs. applied strains and (**b**) Gauge factor vs. applied strains.

Based on previous studies, 3.2 W induced LIG-PEI was used for the bending test, which was carried out with four different strains (0.5, 1, 3, and 5%); bending–releasing cycles were demonstrated. The change in the relative resistance is shown in Figure 8a. An identical resistance change occurs in the sensor, where the strain increases/decreases, and the sensor resistance increases/decreases. Due to the LIG-PEI sensitivity, the sensor responds differently under the cycles of bending/deformation, which shows the good durability and stability of the sensor, as shown in Figure 8. The LIG-PEI suffers the most deformation at the enormous strain (5%), and the insert in Figure 8a shows the details of the strain relaxation. As shown in Figure 8b, the GF of the LIG-PEI sensor was 17 at 0.5% strain, and the highest GF was 141.36 at 5%. This shows that the LIG-PEI is more sensitive than the reported LIG-PI strain sensors, especially the GF and applied strains [18,44].

Overall, this work exploring co-processing and fabrication methods helps to improve the manufacturability/design, repeatability with lesser defects, and scalability of a material. It also emphasizes (1) in situ processing for deposition and irradiation, (2) precision deposition of 350 microns, and (3) combinatorial surface characterization and roughness to explain the improved performance of the processed strain sensor. The demonstrated unique ability of the printable polymer material (using the fused filament fabrication AM technique) with certain desired thicknesses, orientations, and techniques is readily available for further processing (known to be laser processing) without any chemical modification or surface treatment. In addition, the characterized piezo resistivity of the LIG was helpful in understanding the process–structure–property relationship [45]. Significantly, the use of multi-lasing indicates fewer defects in the graphitic structure, and the surface roughness of LIG serves as the electrical interconnection during strain deformation. Moreover, the surface deformation intensity was characterized by electrical measurements to measure the sensitivity performance [46]. Hence, the piezo resistivity of the LIG was studied at a low power of 3.2 W, and the highest amount of deformation of LIG-PEI observed was at the enormous strain of 5% to exhibit the gauge factor (GF) of the sensor. The sensor GF was 17 at 0.5% strain, and the highest GF was 141.36 at 5%. This shows that the LIG-PEI is more sensitive even at 6.6 μm a roughness and 3.2 W laser power.

## 4. Conclusions

In summary, a 3D-printing technique has been used with laser processing as a hybrid processing and co-production method to fabricate flexible, innovative components and for the purpose of sensor-monitoring deformation. The formation of graphitic structures can be prepared on the surface of 3D-printed PEI by controlling the laser irradiation conditions. The morphology and piezo resistivity of the LIG were studied to establish the related process–structure–property relationship. The LIG-PEI sensor shows high sensitivity when subjected to the mechanical test. It is demonstrated as a strain gauge for printable structural self-monitoring flexible sensors of polymeric composite. As shown in this work, the unique performance of LIG using the AM fabrication technique with laser processing can be highly valuable in different industrial sectors for producing customized printable, on-site, and flexible electronics.

## Figures and Tables

**Table 1:** The process parameters of the high-precision printer.

Parameters	Layer Thickness (mm)	Nozzle Size (μm)	Print Speed (mm/s)	Layer Height (mm)	Raster Angle (°)
Layer(n)	0.35	350	15	0.3	90

**Table 2:** List of roughness data and cross-sectional area of LIG at different laser power levels.

Laser Power (W)	Mean Roughness Ra (μm)	LIG Cross-Sectional Area (μm)
3.2	6.6 ± 1.06	56.3 ± 6.3
3.4	6.71 ± 1.17	95.5 ± 2.4
3.6	7.55 ± 1.55	177.0 ± 3.0
3.8	8.71 ± 1.07	208.0 ± 10.0
4.0	11.41 ± 1.14	220.6 ± 18.4

## Data Availability

The data used to support the findings of this study are available from the corresponding author upon request.
